# Carbon–Phosphorus Coupling Governs Microbial Effects on Nutrient Acquisition Strategies by Four Crops

**DOI:** 10.3389/fpls.2022.924154

**Published:** 2022-07-05

**Authors:** Deshan Zhang, Yuqiang Zhang, Zheng Zhao, Sixin Xu, Shumei Cai, Haitao Zhu, Zed Rengel, Yakov Kuzyakov

**Affiliations:** ^1^Institute of Ecological Environment Protection Research, Shanghai Academy of Agricultural Sciences, Shanghai, China; ^2^Shanghai Key Laboratory of Protected Horticultural Technology, Shanghai, China; ^3^Nicholas School of the Environment, Duke University, Durham, NC, United States; ^4^Soil Science and Plant Nutrition, UWA School of Agriculture and Environment, The University of Western Australia, Perth, WA, Australia; ^5^Institute for Adriatic Crops and Karst Reclamation, Split, Croatia; ^6^Department of Soil Science of Temperate Ecosystems, University of Göttingen, Göttingen, Germany; ^7^Department of Agricultural Soil Science, University of Göttingen, Göttingen, Germany; ^8^Peoples Friendship University of Russia (RUDN University), Moscow, Russia; ^9^Institute of Environmental Sciences, Kazan Federal University, Kazan, Russia

**Keywords:** root morphological and exudation traits, root–microbe interaction, fungi, bacteria, phosphate-solubilizing microorganisms, crop P-use efficiency

## Abstract

Plants adjust root morphological and/or exudation traits in response to phosphorus (P) mobilization mediated by microorganisms. We hypothesized that straw application coupled with P fertilization would influence microbial P and then root nutrient-acquisition strategies related to crop growth. Root morphological (length and average diameter) and exudation traits (acid phosphatase and carboxylates) of *Brassica chinensis*, *Solanum lycopersicum, Lactuca sativa*, and *Vigna unguiculata* in response to microbial P dynamics were characterized in no-P and P-fertilized soil with/without straw addition. Straw addition increased the growth of fungi and bacteria, stimulating microbial P immobilization at day 24. The high microbial abundance was associated with four tested crops having short roots in straw-amended compared with no-straw soil at day 24. In straw-amended soil, *B. chinensis* and *S. lycopersicum* shifted toward root P-acquisition strategies based on fast elongation and strong carboxylate exudation from days 24 to 40. Such effective root P-acquisition strategies together with microbial P release increased shoot P content in *S. lycopersicum* in straw-amended compared with those without straw at day 40. Conversely, *L. sativa* and *V. unguiculata* produced short roots in response to the stable (or even increased) microbial P after straw addition till day 40. In straw-amended soil, high P application stimulated root elongation and carboxylate exudation in *L. sativa* and *V. unguiculata*, whereas carboxylate exudation by *S. lycopersicum* was decreased compared with the straw-amended but non-fertilized treatment at day 40. In summary, root P-acquisition strategies in response to microbial P differed among the tested crop species. Phosphorus fertilization needs to be highlighted when returning straw to enhance P-use efficiency in vegetable cropping systems.

## Introduction

Low availability of phosphorus (P) is often limiting plant growth in most soils (Hinsinger, 2001; Shen et al., 2011). Plant species adopt specific root morphological and/or exudation traits to enable efficient P acquisition (Lambers et al., 2006; Wen et al., 2019, 2022). For example, nutrient-acquisitive species with thin roots show rapid root elongation and explore a large soil volume to adapt to low P availability and enhance P acquisition (Li et al., 2017; Wen et al., 2017, 2019). In contrast, some crops with a slow root growth rate demonstrated intensive exudation of P-mobilizing compounds, such as carboxylates and acid phosphatase, in response to P deficiency (Wen et al., 2019, 2020; Koester et al., 2021). Microbial biomass turnover mediated by the growth/death of microorganisms, including phosphate-solubilizing microorganisms (PSM), is critical in increasing soil P availability (Bi et al., 2020; Huang et al., 2021; Raymond et al., 2021). Root morphological and/or exudation traits influencing P acquisition would differ in response to microbial P mobilization and among crop species.

Organic amendments, *via* providing available carbon (C) to microorganisms, trigger the growth of bacteria and fungi, stimulating P immobilization initially, followed by P release from necrotic microbial biomass (Jing et al., 2017; Chen et al., 2019). The dynamic growth and then death (P immobilization and then release) underpin a balance between root morphological and exudation traits related to high P-use efficiency (Marschner et al., 2011; Richardson et al., 2011). For instance, the high microbial abundance leads to intensive root–microbial competition and causes slow growth and short length of roots, suggesting P-conservative strategies underlying increased crop P utilization efficiency (Marschner et al., 2011; Richardson and Simpson, 2011; Cortois et al., 2016). In contrast, P release from necrotic microbial biomass governs acquisitive root morphological and/or exudation traits to enable crop P uptake (Richardson et al., 2011; Ma et al., 2018; Chen et al., 2019). Because of the interspecific variation in root P-acquisition strategies (Wen et al., 2019, 2022), clarifying the dynamics in microbial effects on root morphological and exudation traits among diverse crop species will elucidate the rhizosphere mechanisms underlying root–microbe interactions that influence crop P nutrition and growth.

Phosphorus fertilization increases microbial growth and abundance, driving a high flux of P into microbial biomass when sufficient amounts of available C are present (Chen et al., 2019; Teste et al., 2021). Given that root–microbe competition intensifies due to microbial growth after organic amendment, supplementing available P *via* fertilization would diminish microbial competition and drive the shifts toward acquisitive root strategies (Marschner et al., 2011; Richardson et al., 2011; Ma et al., 2018; Lynch, 2019). The high P release from necrotic microbial biomass as well as direct supplementation of available P expands the size of plant-available P pool in the P-fertilized compared with non-fertilized soil. Among species, crops with diverse types of root system exhibit distinct morphological and exudation plasticity in response to contrasting soil P conditions (Wen et al., 2019). Nevertheless, only very few studies investigated dynamics of root morphological and/or exudation traits related to crop P acquisition depending on the addition of organic materials and P fertilization.

Organic amendment is important to facilitate crop production because of stimulating microorganisms to decompose organic materials and enhance nutrient cycling (Luo et al., 2019; Huang et al., 2021). Such effects are significant for vegetable crops because the addition of organic materials can supply C to microorganisms in soils containing luxury P, but limited C (Yan et al., 2013), thus activating microbial growth and triggering microbially mediated P cycling (Čapek et al., 2018; Soong et al., 2020; Koester et al., 2021). Increasing microbially mediated P bioavailability stimulates fast and deep root growth (Ma et al., 2018; Lynch, 2019; Zhang et al., 2020).

Because a root diameter is one of the significant traits influencing the effects of microbial turnover to crop P acquisition (Hodge et al., 2000), we selected *Brassica chinensis* (*B. chinensis*), *Solanum lycopersicum* (*S. lycopersicum*), *Lactuca sativa* (*L. sativa*), and *Vigna unguiculata* (*V. unguiculata*) with distinct average root diameters as the targeted crops to investigate crop growth potential as influenced by microbial P mobilization. In order to clarify the mechanisms underlying root–microbe interactions related to crop P acquisition, root morphological and exudation traits of the four crops in response to microbial P were characterized in a pot experiment with various straw and P additions.

Specifically, we tested the following hypotheses: (1) Wide interspecific variation of root morphological and/or exudation traits exists when *B. chinensis*, *S. lycopersicum*, *L. sativa*, and *V. unguiculata* adapt to microbial growth/death accompanied by P immobilization/release in straw-amended compared with soil without straw and (2) P fertilization plays a major role in mediating root–microbe interactions, influencing benefits of microbial P mobilization to crop P acquisition and growth.

## Materials and Methods

### Experimental Setup

There were 16 treatment combinations of P supply (no-P and 100 mg P kg^–1^ soil) and straw (with and without), together with four common vegetable crops (*B. chinensis*, *S. lycopersicum*, *L. sativa*, and *V. unguiculata*) to examine the microbial influence on root morphological and/or exudation traits. Two sampling times (24 and 40 days) were set to assess the dynamics of root P-acquisition strategies in response to microbial P immobilization and mobilization after straw addition. Each treatment included four replicates for each sampling time. Therefore, 128 pots were arranged in the experiment.

Soil for the pot experiment was collected from the 0- to 20-cm depth of the paddy soil at Zhuanghang Experimental Station of Shanghai Academy of Agricultural Sciences (30°53′N, 121°22′E) in Shanghai, China. The collected soil was air-dried and passed through a 2-mm sieve. The soil was Gleysol with a bulk density of 1.4 g cm^–3^, 12.3 g kg^–1^ organic C, 1.1 g kg^–1^ total nitrogen (N), 0.8 g kg^–1^ total P, 43 mg kg^–1^ mineral N (NO_3_^–^ + NH_4_^+^), 13 mg kg^–1^ NaHCO_3_-extractable P, 114 mg kg^–1^ NH_4_Ac-extractable potassium (K), and pH of 6.8 (soil/water = 1:5). The methods were according to Bao (2013). Each pot (24 cm diameter at the top, 11 cm at the bottom, and 13 cm high) was filled with 2.5 kg of air-dried soil.

Rice straw collected from farmers after harvesting was oven-dried to constant weight, crushed in a pulverizer, and passed through a 2-mm sieve. Straw was digested in a mixture of K_2_Cr_2_O_7_–H_2_SO_4_ to measure organic C (Bao, 2013). Total N, P, and K were determined by digesting 0.5 g of crushed straw using a mixture of 5 mL of concentrated sulfuric acid and 8 mL of 30% v/v H_2_O_2_ (Bao, 2013). The straw contained 306 g organic C kg^–1^, 4.4 g N kg^–1^, 0.75 g P kg^–1^, and 11.8 g K kg^–1^. The addition of straw with the high C/P ratio (408:1) would result in microbial C mineralization being limited by P, thus requiring P uptake from soil to maintain the optimal microbial C/P ratio ranging from 42:1 to 60:1 (Cleveland and Liptzin, 2007; Xu et al., 2013).

To ensure that the supply of other nutrients was adequate for the plant growth, the soil was fertilized with basal nutrients at the following rates (mg kg^–1^ soil): Ca(NO_3_)_2_⋅4H_2_O, 2250; K_2_SO_4_, 267; MgSO_4_⋅7H_2_O, 87; Fe-EDTA, 11.7; MnSO_4_⋅H_2_O, 13.3; ZnSO_4_⋅7H_2_O, 20; CuSO_4_⋅5H_2_O, 4; H_3_BO_3_, 2.7; and Na_2_MoO_4_⋅5H_2_O, 0.33. Phosphorus in the high-P treatment was applied as Ca(H_2_PO_4_)_2_⋅H_2_O. Mineral nutrients together with 33 g of rice straw (thus adding 4 g C kg^–1^ soil) were mixed thoroughly with soil and packed into the pots.

### Plant Growth

Based on the popular genotype in the south of China, the genotypes of the four crop species were *B. chinensis* L. Xiaqing3, *S. lycopersicum* L. Shenfen16, and *L. sativa* L. Shenxuan4 (provided by Horticultural Institute of Shanghai Academy of Agricultural Sciences), and *V. unguiculata* L. Fengchan8 was provided by Horticultural Institute of Guangdong Academy of Agricultural Sciences. Seeds were surface-sterilized with 10% v/v H_2_O_2_ for 20 min, rinsed with deionized water, soaked in CaSO_4_-saturated solution for 12 h, and then germinated in Petri dishes on wet filter paper at 22°C for 2 days. Four seedlings were planted per pot and later thinned to two plants.

The experiment was conducted in a sunlight glasshouse at Zhuanghang Experimental Station of Shanghai Academy of Agricultural Sciences. Glasshouse temperature was maintained at 21–25°C during the day and 13–16°C at night. We ventilated the glasshouse every day to remain natural humidity. The pots were arranged in a completely randomized design and were re-randomized weekly. The plants were gently watered every day by weight to maintain field capacity (30%, v/v) and were grown for 40 days.

### Harvest and Measurements

In a preliminary experiment from April to May in the year of 2017, the dynamics of CO_2_ emissions indicated that the peak rate of mineralization of rice straw occurred 20 days after addition, with 13% of total straw-derived CO_2_–C released at that time (see also Zhu et al., 2018). Hence, plants were harvested at 24 days (after the maximum initial flush of C) and 40 days after sowing (prolonged release of C at a low rate; see also Zhu et al., 2018) and were separated into shoots and roots.

### Shoot P Content and Root Traits

The shoots of *B. chinensis*, *S. lycopersicum*, *L. sativa*, and *V. unguiculata* were oven-dried at 105°C for 30 min and then at 65°C to constant weight for shoot biomass determination. Roots were carefully brushed, removed from the soil, and gently shaken to remove the loosely adhering soil (considered to be bulk soil), with the soil tightly adhering to roots being defined as rhizosphere soil. Roots with rhizosphere soil were then transferred to a vial containing 50 mL of 0.2 m*M* CaCl_2_ and gently shaken to dislodge the rhizosphere soil (Pearse et al., 2007). Care was taken to minimize root damage.

A subsample (8 mL) of the rhizosphere soil suspension was filtered immediately for the determination of rhizosphere carboxylates by high-performance liquid chromatography (modified from Shen et al., 2003). Two drops of microbial inhibitor Micropur (Sicheres Trinkwasser, Munich, Germany) at 0.01 g L^–1^ were added before storage at –20°C until analysis of carboxylates. When measuring, the filter liquor was passed through cation and then anion exchange resin columns. The eluant was concentrated in a rotary evaporator. The residue was dissolved in dilute HClO_4_ solution with pH of 2.1 and then separated on a Bio-Rad Aminex HPX-87h sulfonic column with eluent containing 5 mM L^–1^ H_2_SO_4_ at 50°C at a flow rate of 0.5 mL min^–1^. Carboxylates were measured spectrophotometrically at 210 nm. Total carboxylates in the rhizosphere soil were analyzed according to Cawthray (2003).

The activity of acid phosphatase in the rhizosphere soil was measured according to Neumann (2006). Two 0.5-mL aliquots of soil suspension were transferred into 2-mL Eppendorf tubes, with 0.4 mL sodium acetate buffer and 0.1 mL *p*-nitrophenyl phosphate (NPP) substrate added. One tube was supplemented with 0.5 mL of 0.5 M NaOH and then incubated at 30°C for 60 min, whereas the other tube was first incubated at 30°C for 60 min, and then, the reaction was terminated by adding 0.5 mL of 0.5 M NaOH. Absorption of the two samples was measured spectrophotometrically (UVmini-1240; Shimadzu, Kyoto, Japan) at 405 nm.

After dislodging the rhizosphere soil, roots were placed in an icebox for transport to the laboratory and were then rinsed with tap water. No nodule was found in *V. unguiculata* roots. The roots were scanned on an EPSON root scanner at 400 dot-per-inch resolution (Epson Expression 1600 pro, Model EU-35, Tokyo, Japan). The total root length (TRL) and average root diameter (RD) were analyzed by Win-RHIZO software (Regent Instruments Inc., Sainte-Foy, Quebec, QC, Canada).

The dried shoots were crushed in a pulverizer and passed through a 2-mm sieve. Phosphorus concentration in shoots was determined by digesting 0.5 g of crushed shoots using a mixture of 5 mL of concentrated sulfuric acid and 8 mL of 30% v/v H_2_O_2_ (Bao, 2013). Shoot P was analyzed by the molybdovanadophosphate method spectrophotometrically (UVmini-1240; Shimadzu, Kyoto, Japan) at 450 nm (Johnson and Ulrich, 1959).

### Microbial Traits

At each harvest, two samples of fresh bulk soil from each pot were collected, thoroughly mixed, and grounded to pass through a 2-mm sieve. One sample of the fresh soil was used for the determination of microbial biomass C (MBC) and P content (MBP). The microbial C and P contents were determined using the chloroform fumigation–extraction method (Brookes et al., 1982; Wu et al., 1990). In brief, fresh soil equivalent to 10 g of air-dried soil was fumigated with 100 mL of ethanol-free CHCl_3_ at room temperature for 48 h. Simultaneously, another sample of fresh soil was treated in the same way, but without ethanol-free CHCl_3_. Organic C was extracted from the fumigated and non-fumigated samples in 40 mL of 0.5 M K_2_SO_4_ and was measured using a TOC/TN analyzer (Multi N/C 2100S, Analytik Jena AG, Jena, Germany). The value of 0.45 was used for the fraction of biomass C mineralized (Wu et al., 1990).

For MBP analysis, samples of fresh bulk soil equivalent to 5 g of air-dried soil were weighed into crucibles and fumigated in a desiccator as per the methods for measuring MBC detailed above. Dissolved P was extracted from the fumigated and non-fumigated samples using a solution of 0.5 M NaHCO_3_ with a 1:10 soil-to-solution ratio (Morel et al., 1996), and P was determined using the molybdenum blue assay (Murphy and Riley, 1962). The correction for adsorption of P during fumigation was made by simultaneously equilibrating non-fumigated soil with a series of P-containing standard solutions followed by extraction with NaHCO_3_ (Morel et al., 1996). The amount of microbial P was estimated by using a Kp factor of 0.4 (Brookes et al., 1982).

The bulk soil samples for examining quantity of genes in bacterial (*16S* rRNA) and fungal (*ITS*) microbiome as well as phosphate-solubilizing functional microbes encoding *phoD* and *pqqC* genes were stored at –80°C until DNA extraction and quantification. Copy numbers of the *16S* rRNA (Janssen, 2006; Luo et al., 2020) and *ITS* (Anderson and Parkin, 2007; Luo et al., 2020) genes as well as the *phoD*-encoding phosphomonoesterase facilitating organic P mineralization (Fraser et al., 2015; Luo et al., 2019) and the *ppqC*-encoding pyrroloquinoline-quinone synthase catalyzing inorganic P dissolution (Zheng et al., 2019; Bi et al., 2020) were measured in three replicates in each sample. We extracted total genomic soil DNA from 0.25 g of soil using a DNeasy Power Soil DNA Isolation Kit (MoBio Laboratories, Carlsbad, CA, United States) according to the manufacturer’s protocols, and stored the extracted DNA at –20°C for subsequent quantitative PCR (qPCR) quantification.

The copies of *16S* rRNA, *ITS*, *phoD*, and *pqqC* genes in bulk soil were measured according to Jing et al. (2017). Extracted DNA was examined with a NanoDrop spectrophotometer (NanoDrop ND-2000c Technologies, Wilmington, DE, United States) and visually checked on agarose gel (1%) before qPCR quantification. Quantitative PCR was carried out using SYBR Premix ExTaqTm Kit (Takara Biotech, Dalian, China). The primers and amplification protocol of genes are listed in Supplementary Table 1. The 25-μL reaction mixture contained 12.5 μL of SYBR matrix, 1 μL primer sets, 2.5 μL template DNA, and 8 μL ultrapure water. A single clone containing the correct insert was grown in Luria–Bertani media, and plasmid DNA was then extracted, purified, and quantified. A tenfold series of dilution of the plasmid DNA was then carried out to generate a standard curve covering six orders of magnitude from 10^3^ to 10^11^ copies of the template per assay. Blanks were always run with water instead of DNA extract. The final gene quantities were obtained by calibrating against total DNA concentrations extracted from the soil.

### Statistical Analyzes

Analysis of variance (ANOVA) was conducted using SPSS version 26.0 (IBM SPSS Inc., Chicago, IL, United States) after normality test of the data. Significant differences among means were separated by Tukey’s test at 5% probability (*p* ≤ 0.05). Crop P content and shoot biomass, root morphological and exudation traits as well as microbial traits were subjected to two-way ANOVA to assess the effects of straw, soil P supply, and their interaction at days 24 and 40 separately. The Student *t*-test (*p* ≤ 0.05) was used to test the difference in each parameter between days 24 and 40.

Principal component analysis (PCA) was performed with the R “prcomp” function to test the difference in microbial effects on root morphological and exudation traits between with and without P fertilizer soils at 24 and 40 days. We used PCA to determine the multivariate ordination of the six soil microbial traits (MBC and MBP, bacterial and fungal microbial abundance as well as phosphate-solubilizing microorganisms in terms of *phoD* and *pqqC* gene abundance) with four root P-acquisition traits (total root length and average diameter as well as rhizosphere carboxylates and acid phosphatase) separately in the *B. chinensis*, *S. lycopersicum*, *L. sativa*, and *V. unguiculata* treatments. PCA plots were drawn with the FAC-TOEXTRA package in R (Kassambara and Mundt, 2017). Furthermore, we calculated the *post hoc* Spearman’s rank correlations to assess relationships between individual root traits and the six soil microbial traits.

## Results

### Shoot Biomass Accumulation and P Acquisition by the Four Crops

**FIGURE 1:**
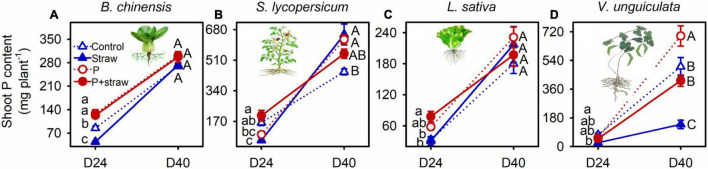
Effects of straw addition and P fertilization on shoot P content in *B. chinensis* **(A)**, *S. lycopersicum* **(B)**, *L. sativa* **(C)**, and *V. unguiculata* **(D)** at days 24 and 40. Control: no fertilizer P or straw; straw: no P but with straw addition; P: fertilizer P at 100 mg P kg^–1^ soil; P + straw: fertilizer P at 100 mg P kg^–1^ soil together with straw addition. D24: 24 days after straw addition, and D40: 40 days after straw addition. Data are means ± SE (*n* = 4). For each crop species, the lowercase letters denote significant differences (*p* ≤ 0.05) among treatments without P or straw, with straw but no fertilizer P, with fertilizer P but no straw, and with fertilizer P and straw at day 24, and the capital letters denote significant differences (*p* ≤ 0.05) among the four treatments at day 40. Significant difference (*p* ≤ 0.001) in shoot P content of the four crops between days 24 and 40 was detected using Student’s *t*-tests (see Supplementary Table 3).

Straw addition and P fertilization influenced shoot biomass and P acquisition by *B. chinensis*, *S. lycopersicum*, and *V. unguiculata*, but not *L. sativa*, at day 24 (Figure 1 and Supplementary Tables 2, 3). Straw addition caused the low shoot biomass and P content in *B. chinensis* in no-P soil with compared to without straw amendment at day 24 (Figure 1A and Supplementary Tables 2, 3). Shoot biomass and P content in *S. lycopersicum* were decreased due to straw addition compared with the no-straw treatments regardless of P supply at day 24. Conversely, crop P acquisition by *S. lycopersicum* in no-P soil was increased after straw addition at day 40 (Figure 1B). Biomass accumulation and P acquisition by *V. unguiculata* were lower in the straw-amended than no-straw soil in both with and without P fertilizer at days 24 and 40 (Figure 1D). In straw-amended soil, high P application increased shoot biomass and shoot P content of *V. unguiculata* at day 40 when compared with non-fertilized crops (Figure 1D).

### Root P-Acquisition Strategies of the Four Crops

**FIGURE 2:**
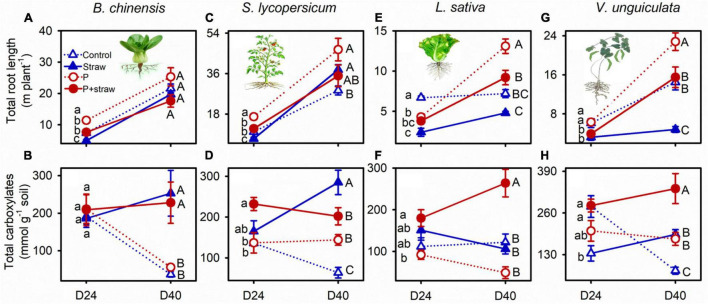
Effects of straw addition and P fertilization on root length and exudation of carboxylates in the rhizosphere of *B. chinensis* **(A,B)**, *S. lycopersicum* **(C,D)**, *L. sativa* **(E,F)**, and *V. unguiculata* **(G,H)** at days 24 and 40. Control: no fertilizer P or straw; straw: no P but with straw addition; P: fertilizer P at 100 mg P kg^–1^ soil; P + straw: fertilizer P at 100 mg P kg^–1^ soil together with straw addition. D24: 24 days after straw addition, and D40: 40 days after straw addition. Data are means ± SE (*n* = 4). For each crop species and a given parameter, the lowercase letters denote significant differences (*p* ≤ 0.05) among treatments without P or straw, with straw but no fertilizer P, with fertilizer P but no straw, and with fertilizer P and straw at day 24, and the capital letters denote significant differences (*p* ≤ 0.05) among the four treatments at day 40.

Straw together with P application influenced root P-acquisition strategies of the four crops (Supplementary Table 2). *B. chinensis* produced short thick roots in response to straw addition in the no- and high-P soils at day 24 and modified the root traits toward strong exudation of carboxylates and acid phosphatase at day 40 (Figures 2A,B and Supplementary Tables 4, 5). Straw addition was associated with short roots in *S. lycopersicum* regardless of P supply at day 24; however, *S. lycopersicum* shifted root traits toward high root elongation accompanied by strong carboxylates and acid phosphatase exudation at day 40 in soil without P fertilizer (Figures 2C,D). Both *L. sativa* and *V. unguiculata* produced short roots in response to straw addition regardless of P fertilization at days 24 and 40 (except *L. sativa* in the high-P soil at day 24; Figures 2E,G). High P application in the straw-amended soil increased root length and exudation of carboxylates by *L. sativa* and *V. unguiculata* but decreased carboxylate exudation by *S. lycopersicum* compared with the no-P supply at day 40 (Figures 2D–H).

### Microbial Biomass and Functionality

Straw addition stimulated bacterial and fungal proliferation at day 24 (except fungi in the no-P soil in which *S. lycopersicum* and *V. unguiculata* grew), independent of P fertilization (Supplementary Table 6). The abundance of phosphate-solubilizing microorganisms encoding *phoD* gene in the no-P soil (as well as in the P-fertilized soil with *B. chinensis* and *S. lycopersicum*) and the functional microorganisms encoding *pqqC* gene in the P-fertilized soil was increased after straw addition (Supplementary Table 7). The abundance of bacteria and fungi as well as phosphate-solubilizing functional microbes encoding *phoD* and *pqqC* genes remained stable (or even increased) in the straw-amended soil regardless of P fertilization from days 24 to 40 (Supplementary Tables 6, 7).

**FIGURE 3:**
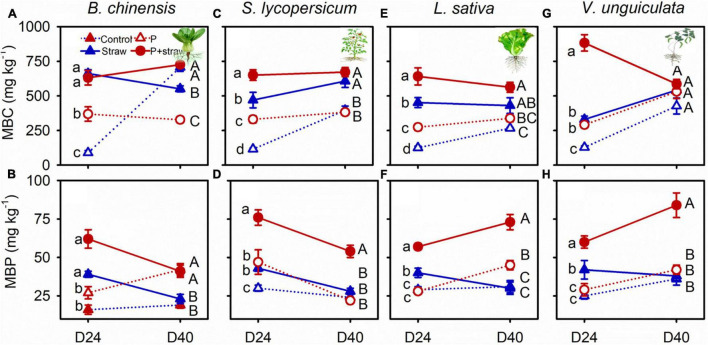
Effects of straw addition and P fertilization on MBC (microbial biomass C) and MBP (microbial biomass P) in soil with *B. chinensis* **(A,B)**, *S. lycopersicum* **(C,D)**, *L. sativa* **(E,F)**, and *V. unguiculata* **(G,H)** after days 24 and 40 days. Control: no fertilizer P or straw; straw: no P but with straw addition; P: fertilizer P at 100 mg P kg^–1^ soil; P + straw: fertilizer P at 100 mg P kg^–1^ soil together with straw addition. D24: 24 days after straw addition, and D40: 40 days after straw addition. Data are means ± SE (*n* = 4). For each crop species and a given parameter, the lowercase letters denote significant differences (*p* ≤ 0.05) among treatments without P or straw, with straw but no fertilizer P, with fertilizer P but no straw, and with fertilizer P and straw at day 24, and the capital letters denote significant differences (*p* ≤ 0.05) among the four treatments at day 40.

At day 24, the amounts of microbial biomass C and P were high in the P-fertilized and non-fertilized soils compared to without straw amendment regardless of crop species (Figure 3 and Supplementary Table 8). Under *B. chinensis* and *S. lycopersicum*, microbial P decreased in the straw-amended soil regardless of P fertilization (Figures 3B,D and Supplementary Table 8). Conversely, microbial P remained stable in straw soil but increased in the straw and P soil in which *L. sativa* and *V. unguiculata* grew from days 24 to 40 (Figures 3F,H and Supplementary Table 7).

### Multivariate Coordination

**FIGURE 4:**
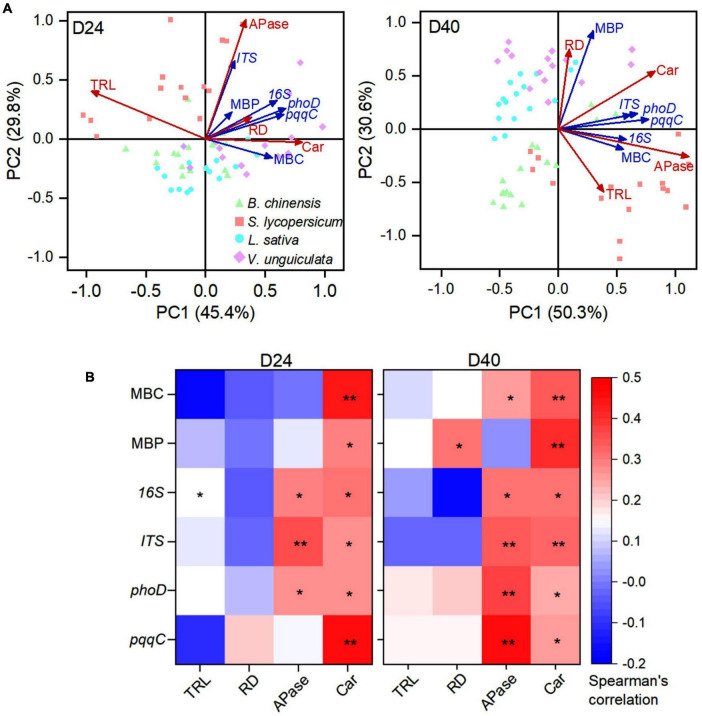
**(A)** Principal component analysis (PCA) and **(B)** spearman’s rank correlation matrices between four root traits and six soil microbe traits *B. chinensis*, *S. lycopersicum*, *L. sativa*, and *V. unguiculata* after 24 and 40 days. D24: 24 days after straw addition, and D40: 40 days after straw addition. Root trait abbreviations: TRL, total root length; RD, average root diameter; Car, concentration of carboxylates in the rhizosphere soil; and APase, acid phosphatase activity in the rhizosphere soil. Microbial trait abbreviations: MBC: microbial biomass C content; MBP: microbial biomass P content; *16S*: abundance of bacteria; *ITS*: abundance of fungi; *phoD*: abundance of *phoD* gene; and *pqqC*: abundance of *pqqC gene*. Asterisks in **(B)** indicate statistical significance (**, *p* ≤ 0.01; *, *p* ≤ 0.05).

The abundance of bacteria and fungi that included phosphate-solubilizing microorganisms encoding *phoD* and *pqqC* genes was positively associated with exudation of carboxylates at days 24 and 40 (Figure 4). The total root length was governed by a high abundance of bacteria in soil at day 24 after straw addition. Microbial C was positively correlated with exudation of carboxylates and acid phosphatase, and microbial P was positively associated with carboxylate exudation at day 40. Microbial P was negatively correlated with the total root length (*p* = 0.068), but positively correlated with a root diameter at day 40. Specifically, the root diameter and microbial P were scattered in the direction of *L. sativa* and *V. unguiculata*, whereas the total root length was located in the direction of *B. chinensis* and *S. lycopersicum* at day 40.

## Discussion

### Straw Application Coupled With P Fertilization Increased Microbial P

The abundance of bacteria and fungi including phosphate-solubilizing microbial groups encoding *phoD* and *pqqC* genes was increased at day 24 after straw addition (Supplementary Tables 6, 7). This was mainly because organic materials supplied C to stimulate the growth of bacteria and fungi that included phosphate-solubilizing functional groups (Jing et al., 2017; Chen et al., 2019; Huang et al., 2021). Microbial growth immobilized orthophosphate in soil (Chen et al., 2019), resulting in high microbial P in straw-amended compared with no-straw soil at day 24 (Figure 3 and Supplementary Table 8). Microbial cells turn over more rapidly than plant roots do, therefore releasing nutrient back into the soil, whereas plants are able to retain captured nutrient to a greater extent (Hodge et al., 2000). Although organic C is depleted in soil over long time, carboxylates and acid phosphatase represent C-containing substrates to rhizosphere microbes (Bicharanloo et al., 2020; Ding et al., 2021), underpinning stable microbial biomass C in the straw-amended compared with no-straw soil from days 24 to 40 (Figures 2, 3, 4 and Supplementary Tables 5, 8). Phosphorus acquisition in *B. chinensis* and *S. lycopersicum* was associated with a decline in microbial P in straw-amended soil from days 24 to 40 (Figures 1A,B, 3B,D and Supplementary Table 8). In contrast, microbial P remained stable in straw-amended soil under *L. sativa* and *V. unguiculata*, resulting from the slow and small P acquisition in the two crops during days 24 to 40 (Figures 1C,D, 3F,H and Supplementary Table 8).

Phosphorus fertilization maximized microbial P pool when there was sufficient available C (Chen et al., 2019), resulting in high microbial P in soil amended with straw and P compared to that without P fertilizer during the first 24 days (Figures 3B,D,F,H). Because high amounts of available P supplemented *via* fertilization satisfied nutrient demand for crops and microbes from days 24 to 40 (Figure 1), in straw soil microbial P remained high in P-fertilized compared to non-fertilized treatments at day 40 (Figures 3B,D,F,H). The results indicated that straw together with P fertilizer governed high microbial P to determine large crop P acquisition.

### Root P-Acquisition Strategies in Response to Microbial P Differed Among Crops

Microbial growth and P immobilization indicated intensive competition between roots and microbes (Marschner et al., 2011; Richardson et al., 2011). The tested four crops produced short roots in the straw-amended compared with no-straw soil during the first 24 days (Figures 2A,C,E,G), suggesting P-conservative root traits based on slow elongation under microbial growth (McCormack et al., 2015; Cortois et al., 2016). The slow root growth conferred stress resistance by reducing C demand for roots, thereby allowing for great investment in defense traits (e.g., high root tissue density) to tolerate microbial proliferation (Chapin et al., 1993; Valverde-Barrantes et al., 2017). Furthermore, the slow growth of roots indicated crops produced roots with long life span and low turnover rate in order to decrease P loss *via* shed cells and increase nutrient conservation for high use efficiency in shoots (McCormack et al., 2015). Crops, regardless of species, decreased root elongation to avoid microbial competition and conserve P for the growth during the first 24 days after straw addition.

The bacterial abundance remained high in straw-amended compared with no-straw soil *B. chinensis* growing at days 24 and 40 (Supplementary Table 6). Conversely, the fungal abundance was high in straw than no-straw soil at day 24, but became the same level as those in no-straw soil at day 40 (Supplementary Table 6). The results indicated a decline in root-microbe competition in straw-amended soil under *B. chinensis* at day 40 compared with day 24. In straw-amended soil under *S. lycopersicum*, *L. sativa*, and *V. unguiculata*, the bacterial and fungal abundance remained the same level as those without straw added at day 40 (Supplementary Table 6). The results indicated root–microbial competition was weak to determine root traits in straw-amended soil at day 40 compared with day 24.

**FIGURE 5:**
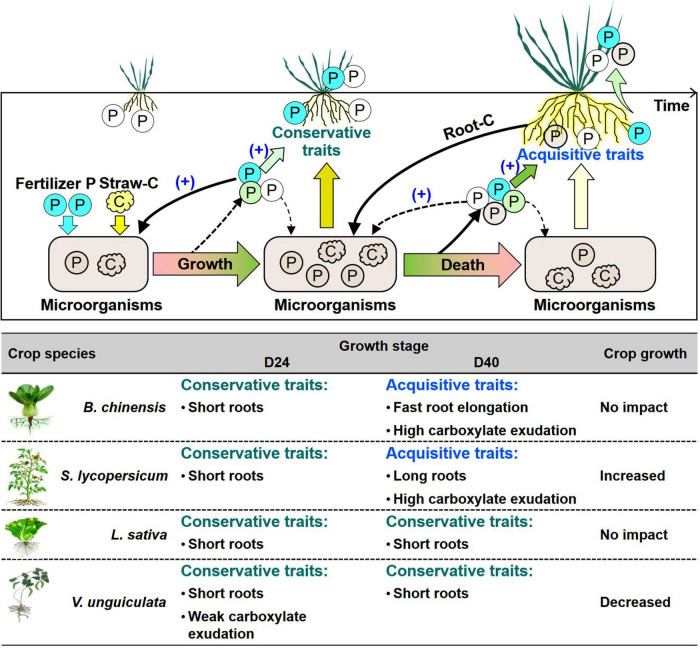
Framework of the root-microbe interactions driven by straw addition and P fertilization as well as the role of these interactions in P uptake by *B. chinensis*, *S. lycopersicum*, *L. sativa*, and *V. unguiculata*. The solid-line black arrows indicate the strong interactions among soil P, microbes, and roots influencing plant growth, whereas the dashed arrows denote the relatively weak effects. The white circles indicate the available P in soil; the blue circles denote the added fertilizer P; and the pink circles represent the processes of P release from microbial biomass to the plant-available P pool, but no supporting data are available so far. The table denotes the changes in root morphological and exudation traits with straw addition in comparison with roots without straw addition after day 24 (D24) and 40 days (D40). A dark green arrow indicates the strong influence of soil P on root traits, whereas a light green one indicates the weak influence. A light brown arrow indicates the strong influence of microbes on root traits, whereas a light yellow one denotes the weak influence. The plus in parentheses indicates the P fertilization increases the role of P in governing root traits related to crop P acquisition and microbial P immobilization.

Microbial P turnover was important in increasing plant-available P (Raymond et al., 2021; Lu et al., 2022). In soil with weak root-microbe competition at day 40, soil P availability became important to determine root P-acquisition strategies (Figure 4). In straw-amended soil, *B. chinensis* and *S. lycopersisucm* produced roots to reach or exceed crops in no-straw soil at day 40, indicating fast growth during days 24 to 40 to compensate the short length at day 24 (Figures 2A,C). Absorptive long roots and strong carboxylate exudation are involved in efficient nutrient acquisition, especially in competition with microorganisms (Lynch, 2019). Crop P content of thick-root species mainly relied on exudation of P-mobilizing compounds under P deficiency (Shen et al., 2011; Wen et al., 2020). Strong carboxylate exudation by *L. sativa* and *V. unguiculata* indicated crops adopted P-acquisitive root traits in straw-amend soil compared with those in no-straw soil at day 40 (Figure 2). In summary, the four tested crops adopted conservative strategies based on short length during the first 24 days after straw addition, whereas each tested crop species adopted specific root P-acquisition strategies in response to microbial P turnover during days 24 to 40 (Figure 5).

Soil P availability was significant to root P-acquisitive traits but differed highly among crop species at day 40 after straw addition (Figure 4). For instance, P fertilization in the straw-amended soil governed fast root elongation and strong exudation of carboxylates by *L. sativa* and *V. unguiculata* at day 40, determining efficiency P-acquisitive traits to recovery from microbial competition (Figures 2E–H). In contrast, when soil P was sufficiency to crops, in straw-amended soil P fertilization diminished exudation of carboxylates and acid phosphatase by *S. lycopersicum* (Figures 2C,D). Root–microbe interactions under straw amendment highly depended on P fertilization (Figure 5).

### Phosphorus Acquisition Underlying Root–Microbe Interactions Determined by Straw Amendment, P Fertilization, and Crop Species

Short roots and microbial P immobilization decreased shoot P content in *B. chinensis* and *S. lycopersicum* in straw compared to those without straw at day 24 (Figures 1A,B). Root elongation accompanied by strong carboxylate exudation together with microbial P release suggested efficient P acquisition and shoot accumulation (Lynch, 2019; Raymond et al., 2021; Lu et al., 2022), such as in *B. chinensis* and *S. lycopersicum* with straw treatments from days 24 to 40 (Figures 2A–2D, 3B,D). Such high crop P acquisition compensated the low shoot P content before day 24, eliciting similar crop P content in *B. chinensis* and even larger shoot P content in *S. lycopersicum* in the straw-amended compared with no-straw soil at day 40 (Figures 1A,B). The short roots and low carboxylate exudation resulted in low shoot P content in *V. unguiculata* in the straw-amended compared with no-straw soil across the experiment, whereas *L. sativa* was influenced little due to having the lowest P demand among the four crops (Figures 1C,D).

A large pool of plant-available P (in the high-P compared with no-P soil) eliminated P limitation imposed by microbially derived P immobilization (Spohn and Widdig, 2017; Chen et al., 2019), underpinning crop P acquisition by the four tested species under microbial competition (Figure 1). Root elongation accompanied by strong exudation of carboxylates indicated high P acquisition by *L. sativa* and *V. unguiculata* in the P + straw compared with the straw without P soil at day 40 (Figure 1D; Lynch, 2019). By contrast, sufficient P decreased carboxylate exudation of *S. lycopersicum* at day 40 (Figures 2C,D), potentially limiting benefits to crop growth from mobilization of microbial P. In conclusion, straw application coupled with P fertilization governed P acquisition (influenced by root–microbe interactions) differently in the four tested crop species.

### Highlights

Crop species, straw amendment, and P fertilization need to be considered simultaneously for high P-use efficiency in vegetable cropping systems. *S. lycopersicum* benefited from microbial P mobilization in no-P soil after straw addition (Figure 2), indicating a high potential of straw-governed microbial P mobilization for increasing P-use efficiency. By contrast, the low shoot P content of thick-rooted *V. unguiculata* highlighted specific management of P fertilization coupled with straw returning needed to be considered to increase crop P acquisition (Figure 2). The match between fertilizer P application and crop species is critical in increasing P-acquisition efficiency and saving fertilizer *via* organic material amendments. Further and detailed research is needed to examine how to maximize the benefits of microbial P mobilization to crop P-use efficiency.

The microbial populations in the rhizosphere and bulk soil can differ significantly (Edwards et al., 2018). It could draw attention to provide adequate justification for disparity of microbes between rhizosphere and bulk soil as well as the role in governing dynamic root-microbe interactions. Mycorrhizal symbiosis is the key player in aiding plant nutrient uptake although the dependency of plants on this symbiosis tends to vary (Wen et al., 2019, 2022). Further studies need to clarify how rhizosphere microbial composition governs tradeoffs among root morphology, exudation, and mycorrhizal symbioses for phosphorus-acquisition strategies. The key microbial taxa or microbe-derived phytohormones are important in influencing root morphological and exudation traits related to crop nutrient acquisition (Huang et al., 2021; Sun et al., 2021; Mamet et al., 2022; Xu et al., 2022). It is necessary to clarify the mechanisms of dynamic root-microbe interactions underlying rhizosphere microbial composition or phytohormones in soils treated with organic material and P fertilization.

## Conclusion

Straw addition triggered the growth of bacteria and fungi, including phosphate-solubilizing functional groups, to increase microbial P. The high abundance of microorganisms was associated with short roots of the four tested species. In straw-amended soil, *B. chinensis* and *S. lycopersicum* shifted from short roots in response to microbial P immobilization to fast root elongation accompanied by strong carboxylate exudation upon release of microbial P. Conversely, after straw addition, *L. sativa* and *V. unguiculata* tended to adopt conservative strategies based on short roots. In straw-amended soil, alleviation of P deficiency *via* fertilization stimulated root growth and carboxylate exudation by *L. sativa* and *V. unguiculata*, whereas efficiency in root P-acquisition by *S. lycopersicum* was decreased compared with the non-fertilized soil. In summary, root P-acquisition strategies in response to microbial P dynamics after straw addition differed among the four crops tested in the present experiment. High-P fertilization may need to be considered together with returning straw to ensure strong crop growth.

## Data Availability Statement

The raw data supporting the conclusions of this article will be made available by the authors, without undue reservation.

## Author Contributions

DZ, SX, SC, and HZ conceived the ideas and collected and analyzed the data. All authors interpreted the data and revised the manuscript.

## Conflict of Interest

The authors declare that the research was conducted in the absence of any commercial or financial relationships that could be construed as a potential conflict of interest.

## Publisher’s Note

All claims expressed in this article are solely those of the authors and do not necessarily represent those of their affiliated organizations, or those of the publisher, the editors and the reviewers. Any product that may be evaluated in this article, or claim that may be made by its manufacturer, is not guaranteed or endorsed by the publisher.
