# Supplementary material for: Carbon–Phosphorus Coupling Governs Microbial Effects on Nutrient Acquisition Strategies by Four Crops
Source: Front Plant Sci. 2022 Jul 5;13:924154. doi: 10.3389/fpls.2022.924154 (PMC9294595; doi:10.3389/fpls.2022.924154)
Supplement: Supplementary file 1 [file Data_Sheet_1.docx]

**Supplementary Information for**

**Carbon-phosphorus coupling governs microbial effects on nutrient acquisition strategies by four crops**

Deshan Zhang^1, 2^, Yuqiang Zhang^3^, Zheng Zhao^1, 2^, Sixin Xu^1, 2^, Shumei Cai^1, 2^, Haitao Zhu^1, 2*^, Zed Rengel^4, 5^, Yakov Kuzyakov^6, 7^

^1^ *Institute of Ecological Environment Protection Research,* *Shanghai Academy of Agricultural Sciences, Shanghai 201403, China*

^2^ *Shanghai Key Laboratory of Protected Horticultural Technology, Shanghai 201403, China*

*^3^ Nicholas School of the Environment, Duke University, Durham, NC, USA*

*^4^* *Soil Science and Plant Nutrition, UWA School of Agriculture and Environment, The University of Western Australia, Perth, WA 6009, Australia*

^5^ *Institute for Adriatic Crops and Karst Reclamation, Split 21000, Croatia*

^6^ *Department of Soil Science of Temperate Ecosystems, Department of Agricultural Soil Science, University of Göettingen, Göttingen 37077, Germany*

^7^ *Peoples Friendship University of Russia (RUDN University), 117198 Moscow; Institute of Environmental Sciences, Kazan Federal University, Kazan 420049, Russia*

**TableS1** Information on amplification of bacterial *16S* rRNA gene, fungal *ITS* region as well as *phoD* and *pqqC* genes.

| Primer set (5’-3’) | Target gene | Amplicon length (bp) | Amplification efficiency | Amplification cycling conditions | References |
| --- | --- | --- | --- | --- | --- |
| Fw (ACTCCTACGGGAGGCAGCAG) | *16S* rRNA | *185* | 96-99% | 40 cycles (95°C 30s, 60°C 5s, 72°C 34s) | Janssen, 2006; Luo et al., 2020 |
| Rv (ATTACCGCGGCTGCTGG) |  |  |  |  |  |
| Fw (TCCGTAGGTGAACCTGCGG) | *ITS* | *300* | 95-100% | 40 cycles (95°C 30s, 60°C 5s, 72°C 34s) | Anderson and Parkin, 2007;  Luo et al., 2020 |
| Rv (CGCTGCGTTCTTCATCG) |  |  |  |  |  |
| Fw (CAGTGGGACGACCACGAGGT) | *phoD* | 371 | 96–103% | 40 cycles (95°C 30s, 60°C 5s, 72°C 34s) | Fraser et al., 2015; Luo et al., 2019 |
| Rv (GAGGCCGATCGGCATGTCG) |  |  |  |  |  |
| Fw (CATGGCATCGAGCAT GCT CC) | *pqqC* | 312 | 97–105% | 40 cycles (95°C 30s, 58°C 5s, 72°C 34s) | Zheng et al., 2019; Bi et al., 2020 |
| Rv (CAGGGCTGGGTCGCCAACC |  |  |  |  |  |

**References**

Anderson, I. C., Parkin, P. I. (2007). Detection of active soil fungi by RT-PCR amplification of precursor rRNA molecules. J Microbiol. Meth. 68, 248-253. doi: 10.1016/j.mimet.2006.08.005

Bi, Q. F., Li, K. J., Zheng, B. X., Liu, X. P., Li, H. Z., and Jin, B. J., et al. (2020). Partial replacement of inorganic phosphorus (P) by organic manure reshapes phosphate mobilizing bacterial community and promotes P bioavailability in a paddy soil. Sci. Total Environ. 703, 134977. doi: 10.1016/j.scitotenv.2019.134977

Fraser, T. D., Lynch, D. H., Bent, E., Entz, M. H., Dunfield, K. E. (2015). Soil bacterial phoD gene abundance and expression in response to applied phosphorus and long-term management. Soil Biol. Biochem. 88, 137-147. doi:10.1016/j.soilbio.2015.04.014

Janssen, P. H. (2006). Identifying the dominant soil bacterial taxa in libraries of *16S* rRNA and *16S* rRNA genes. App. Environ. Microb. 72, 1719-1728. doi: 10.1128/AEM.72.3.1719-1728.2006

Luo, G., Sun, B., Li, L., Li, M., Liu, M., and Zhu, Y., et al. (2019). Understanding how long-term organic amendments increase soil phosphatase activities: insight into *phoD*- and *phoC*-harboring functional microbial populations. Soil Biol. Biochem. 139, 107632. doi: 10.1016/j.soilbio.2019.107632

Luo, G., Xue, C., Jiang, Q., Xiao, Y., Zhang, F., Guo, S., Shen, Q., Ling, N. (2020). Soil carbon, nitrogen, and phosphorus cycling microbial populations and their resistance to global change depend on soil C: N: P stoichiometry. mSystems 5, e00162-00120. doi:10.1128/mSystems.00162-20

Zheng, B. X., Ding, K., Yang, X. R., Wadaan, M. A. M., Hozzein, W. N., Penuelas, J., Zhu, Y. G. (2019. Straw biochar increases the abundance of inorganic phosphate solubilizing bacterial community for better rape (*Brassica napus*) growth and phosphate uptake. Sci. Total Environ. 647, 1113-1120. doi: 10.1016/j.scitotenv.2018.07.454

**Table S2** 2-way ANOVA of the effects of straw addition and P fertilization on crop growth, root morphological and exudation traits as well as microbial traits in treatments with *B. chinensis*, *S. lycopersicum*, *L. sativa*, and *V. unguiculata* at days 24 and 40.

| Measurements | *B. chinensis* | | | | | | *S. lycopersicum* | | | | | | *L. sativa* | | | | | | *V. unguiculata* | | | | | |
| --- | --- | --- | --- | --- | --- | --- | --- | --- | --- | --- | --- | --- | --- | --- | --- | --- | --- | --- | --- | --- | --- | --- | --- | --- |
|  | D24 | | | D40 | | | D24 | | | D40 | | | D24 | | | D40 | | | D24 | | | D40 | | |
|  | P | S | SxP | P | S | SxP | P | S | SxP | P | S | SxP | P | S | SxP | P | S | SxP | P | S | SxP | P | S | SxP |
| SB | ******* | ***** | ***** | ns | ns | ns | ns | ******* | ns | ns | ns | ***** | ****** | ns | ns | ns | ns | ns | ns | ****** | ***** | ******* | ******* | ns |
| SP | ******* | ***** | ***** | ns | ns | ns | ns | ******* | ns | ns | ns | ***** | ****** | ns | ns | ns | ns | ns | ns | ****** | ***** | ******* | ******* | ns |
| TRL | ******* | ******* | ns | ns | ***** | ns | ******* | ****** | ns | ns | ns | ***** | ns | ******* | ****** | ****** | ****** | ns | ns | ****** | ns | ******* | ******* | ns |
| RD | ***** | ****** | ns | ns | ns | ****** | ns | ns | ns | ***** | ns | ns | ns | ns | ns | ns | ns | ns | ns | ns | ns | ns | ns | ns |
| APase | ns | ns | ns | ***** | ****** | ns | ***** | ns | ns | ns | ****** | ****** | ***** | ******* | ns | ns | ****** | ns | ***** | ***** | ns | ******* | ******* | ******* |
| Carboxylates | ns | ns | ns | ns | ****** | ns | ns | ns | ns | ns | ******* | ****** | ns | ****** | ns | ns | ****** | ******* | ns | ****** | ns | ****** | ns | ******* |
| MBC | ****** | ****** | ******* | ******* | ****** | ******* | ns | ******* | ******* | ns | ns | ******* | ns | ****** | ******* | ns | ***** | ******* | ******* | ******* | ******* | ns | ns | ns |
| MBP | ns | ****** | ******* | ns | ******* | ns | ***** | ******* | ******* | ******* | ******* | ******* | ns | ns | ******* | ******* | ******* | ******* | ****** | ****** | ******* | ******* | ******* | ******* |
| Bacteria | ns | ******* | ns | ns | ******* | ns | ns | ******* | ns | ****** | ***** | ***** | ns | ******* | ns | ns | ns | ns | ns | ******* | ns | ns | ns | ns |
| Fungi | ns | ****** | ns | ***** | ***** | ns | ***** | ***** | ns | ns | ***** | ns | ns | ****** | ns | ns | ****** | ns | ***** | ***** | ns | ****** | ******* | ns |
| *phoD* | ns | ***** | ns | ***** | ns | ns | ns | ****** | ns | ns | ns | ns | ns | ***** | ns | ns | ***** | ***** | ns | ****** | ns | ns | ns | ns |
| *pqqC* | ns | ***** | ns | ****** | ns | ns | ***** | ******* | ****** | ns | ns | ns | ns | ****** | ***** | ***** | ****** | ns | ****** | ******* | ******* | ns | ******* | ns |

Note: S: straw addition; P: mineral P fertilization. D24: 24 days after straw addition, and D40: 40 days after straw addition. SB: shoot biomass; SP: shoot P content; TRL: total root length; RD: average root diameter; APase: acid phosphatase; MBC: microbial biomass C; MBP: microbial biomass P; *phoD*: abundance of *phoD* gene; *pqqC*: abundance of *pqqC* gene. Statistically significant values are denoted as: *, p ≤0.05; **, p ≤0.01; and ***, p ≤0.001. ns = non-significant.

**Table S3** Effects of straw addition and P fertilization on shoot biomass and P content in *B. chinensis*, *S. lycopersicum*, *L. sativa*, and *V. unguiculata* at days 24 and 40.

| Treatments | | Shoot biomass  (g plant^-1^) | | Shoot P content  (mg plant^-1^) | |
| --- | --- | --- | --- | --- | --- |
|  |  | D24 | D40 | D24 | D40 |
| *B. chinensis* | Control | 1.04±0.07a | 2.79±0.14A(***) | 85±6y | 269±4X(***) |
|  | Straw | 0.61±0.04b | 2.63±0.16A(***) | 44±2z | 273±4X(***) |
|  | P | 1.03±0.05a | 2.7±0.11A(***) | 128±12x | 301±10X(***) |
|  | P + straw | 1.01±0.13a | 2.43±0.08A(***) | 123±8x | 297±16X(***) |
| *S. lycopersicum* | Control | 2.73±0.25a | 7.73±0.53A(***) | 163±17xy | 445±17XY(***) |
|  | Straw | 1.02±0.13b | 7.98±0.36A(***) | 65±5z | 653±58X(***) |
|  | P | 2.35±0.18a | 8.68±0.51A(***) | 202±30x | 546±26XY(***) |
|  | P + straw | 1.18±0.14b | 7.65±0.21A(***) | 97±12yz | 625±27X(***) |
| *L. sativa* | Control | 0.48±0.04b | 2.25±0.1A(***) | 33±4y | 180±19X(***) |
|  | Straw | 0.43±0.09b | 2.28±0.22A(***) | 31±8y | 217±35X(***) |
|  | P | 0.62±0.08ab | 2.24±0.17A(***) | 58±6xy | 231±20X(***) |
|  | P + straw | 0.82±0.11a | 2.63±0.22A(***) | 78±10x | 197±22X(***) |
| *V. unguiculata* | Control | 1.17±0.14a | 4.53±0.22B(***) | 73±11x | 501±57Y(***) |
|  | Straw | 0.76±0.08b | 2.03±0.22C(***) | 24±4y | 139±26Z(***) |
|  | P | 0.95±0.13ab | 6.1±0.44A(***) | 54±9xy | 694±64X(***) |
|  | P + straw | 1.01±0.05ab | 4.38±0.26B(***) | 49±1xy | 413±36Y(***) |

Note: Control: no fertilizer P or straw; Straw: no P but with straw addition; P: fertilizer P at 100 mg P kg^-1^ soil; P + straw: fertilizer P at 100 mg P kg^-1^ soil together with straw addition. D24: 24 days after straw addition, and D40: 40 days after straw addition. D24: 24 days after straw addition, and D40: 40 days after straw addition. Data are means ± SE (n = 4). For each crop species and a given parameter, the lowercase letters denote significant differences (*p* ≤0.05) among treatments without P or straw, with straw but no fertilizer P, with fertilizer P but no straw, and with fertilizer P and straw at day 24, and the capital letters denote significant differences (*p* ≤0.05) among the four treatments at day 40. For each parameter, t-tests were run to detect significant difference between days 24 and 40: ***, p ≤0.001.

**Table S4** Effects of straw addition and P fertilization on root length and diameter of *B. chinensis*, *S. lycopersicum*, *L. sativa*, and *V. unguiculata* at days 24 and 40.

| Treatments | | Total root length  (m plant^-1^) | | Average root diameter  (mm) | |
| --- | --- | --- | --- | --- | --- |
|  |  | D24 | D40 | D24 | D40 |
| *B. chinensis* | Control | 7.4±0.5b | 21.6±1.4A(***) | 0.17±0.01y | 0.16±0.01Y |
|  | Straw | 4.9±0.4c | 19.7±0.6A(***) | 0.2±0.01x | 0.19±0.01X |
|  | P | 11.4±0.4a | 25.3±2.9A(**) | 0.15±0.01z | 0.18±0.01X(*) |
|  | P + straw | 7.6±1b | 17.6±2A(**) | 0.18±0y | 0.17±0.01X |
| *S. lycopersicum* | Control | 10.1±0.9b | 28.5±2B(***) | 0.25±0.01x | 0.25±0.01X |
|  | Straw | 7±0.7c | 37.7±2.3A(***) | 0.25±0.01x | 0.23±0.01XY |
|  | P | 16.8±1.3a | 46.7±5A(**) | 0.22±0.01x | 0.22±0.02XY |
|  | P + straw | 11.5±1.2b | 35±4.8AB(**) | 0.24±0.01x | 0.2±0.01Y(**) |
| *L. sativa* | Control | 6.7±0.2a | 7.2±0.5BC | 0.26±0.01x | 0.27±0.01X |
|  | Straw | 2.4±0.5c | 4.8±0.2C(*) | 0.28±0.01x | 0.26±0.01X |
|  | P | 4.3±0.2b | 13.1±0.9A(**) | 0.26±0.01x | 0.26±0.01X |
|  | P + straw | 3.8±0.2c | 9.2±0.9B(**) | 0.27±0.01x | 0.25±0.01X |
| *V. unguiculata* | Control | 6.1±0.9a | 14.5±1.6B(**) | 0.37±0.01x | 0.32±0.01X(*) |
|  | Straw | 3.2±0.6b | 4.8±0.6C | 0.37±0.02x | 0.34±0.01X |
|  | P | 6.3±0.4a | 22.8±1.8A(***) | 0.34±0.02x | 0.33±0.01X |
|  | P + straw | 3.9±0.3b | 15.5±2.1B(**) | 0.34±0.03x | 0.32±0.01X |

Note: Control: no fertilizer P or straw; Straw: no P but with straw addition; P: fertilizer P at 100 mg P kg^-1^ soil; P + straw: fertilizer P at 100 mg P kg^-1^ soil together with straw addition. D24: 24 days after straw addition, and D40: 40 days after straw addition. D24: 24 days after straw addition, and D40: 40 days after straw addition. Data are means ± SE (n = 4). For each crop species and a given parameter, the lowercase letters denote significant differences (*p* ≤0.05) among treatments without P or straw, with straw but no fertilizer P, with fertilizer P but no straw, and with fertilizer P and straw at day 24, and the capital letters denote significant differences (*p* ≤0.05) among the four treatments at day 40. For each parameter, t-tests were run to detect significant difference between days 24 and 40: *, p ≤0.05; **, p ≤0.01; ***, p ≤0.001. Absence of asterisks denotes no difference between days 24 and 40.

**Table S5** Effects of straw addition and P fertilization on exudation of acid phosphatase and carboxylates in the rhizosphere soil of *B. chinensis*, *S. lycopersicum*, *L. sativa*, and *V. unguiculata* at days 24 and 40.

| Treatments | | Acid phosphatase  (× 10^3^ PNP h^-1^ g^-1^ soil) | | Total carboxylates  (μmol g^-1^ soil) | |
| --- | --- | --- | --- | --- | --- |
|  |  | D24 | D40 | D24 | D40 |
| *B. chinensis* | Control | 19.4±2.6a | 3.6±1.1BC(**) | 191±20x | 37±7Y(***) |
|  | Straw | 21.5±4.1a | 11.8±1.9A | 186±24x | 253±61X |
|  | P | 20.4±5.8a | 2.4±0.5C(*) | 211±38x | 56±6Y(**) |
|  | P + straw | 13.3±1a | 5.8±1.2B(**) | 209±42x | 228±55X |
| *S. lycopersicum* | Control | 46.2±6.5ab | 11.2±1.4B(**) | 136±4y | 64±13Z(**) |
|  | Straw | 53.7±8.9a | 43.2±2.7A | 165±26xy | 285±30X(*) |
|  | P | 19.2±4.6b | 33.6±5A | 137±25y | 144±13Y |
|  | P + straw | 35.9±9.1ab | 34.5±3.8A | 232±16x | 202±21Y |
| *L. sativa* | Control | 10.5±1.1bc | 1.8±0.4B(***) | 112±21xy | 122±20Y |
|  | Straw | 21.7±3.8a | 7.3±1.8A(*) | 151±24xy | 106±12Y |
|  | P | 4.8±0.6c | 4±0.8B | 92±11y | 49±14Y(*) |
|  | P + straw | 14.7±1.4ab | 7.6±1.1A(**) | 180±20x | 264±33X |
| *V. unguiculata* | Control | 16.4±2.2b | 5.8±1B(**) | 280±34x | 80±11Z(**) |
|  | Straw | 38.1±8.1a | 3.7±0.7B(**) | 134±24y | 193±15Y |
|  | P | 14.1±2.3b | 5±1B(*) | 204±32xy | 179±20Y |
|  | P + straw | 18.2±2.3b | 23.4±2.9A | 283±20x | 336±47X |

Note: Control: no fertilizer P or straw; Straw: no P but with straw addition; P: fertilizer P at 100 mg P kg^-1^ soil; P + straw: fertilizer P at 100 mg P kg^-1^ soil together with straw addition. D24: 24 days after straw addition, and D40: 40 days after straw addition. D24: 24 days after straw addition, and D40: 40 days after straw addition. Data are means ± SE (n = 4). For each crop species and a given parameter, the lowercase letters denote significant differences (*p* ≤0.05) among treatments without P or straw, with straw but no fertilizer P, with fertilizer P but no straw, and with fertilizer P and straw at day 24, and the capital letters denote significant differences (*p* ≤0.05) among the four treatments at day 40. For each parameter, t-tests were run to detect significant difference between days 24 and 40: *, p ≤0.05; **, p ≤0.01; ***, p ≤0.001. Absence of asterisks denotes no difference between days 24 and 40.

**Table S6** Effects of straw addition and P fertilization on abundance of *16S* rRNA and *ITS* genes in the bulk soil in which *B. chinensis*, *S. lycopersicum*, *L. sativa*, and *V. unguiculata* at days 24 and 40.

| Treatments | | *16S* rRNA gene  (× 10^9^ copies g^-1^ soil) | | *ITS* gene  (× 10^6^ copies g^-1^ soil) | |
| --- | --- | --- | --- | --- | --- |
|  |  | D24 | D40 | D24 | D40 |
| *B. chinensis* | Control | 2.8±0.3b | 4.3±0.3B(*) | 3.2±0.2y | 3±0.4Y |
|  | Straw | 11.8±1.5a | 9.2±0.9A | 6.1±0.5x | 4.3±0.6Y |
|  | P | 3.7±0.2b | 5±0.5B(*) | 3.4±0.8y | 4.3±1Y |
|  | P + straw | 11.2±1.2a | 9.7±1A | 7.8±0.4x | 7.2±0.5X |
| *S. lycopersicum* | Control | 3.6±0.2b | 8.9±1.2A(**) | 4.7±0.8y | 5.4±0.8Y |
|  | Straw | 11.3±0.9a | 11±0.4A | 7±1.2xy | 7.8±0.9XY |
|  | P | 3.7±0.3b | 4.4±0.6B | 6.8±0.8y | 4.1±0.8Y |
|  | P + straw | 10.5±1.1a | 10.5±0.4A | 11±1.3x | 9±1.8X |
| *L. sativa* | Control | 4±0.1b | 6.2±0.9AB(*) | 2.6±0.6y | 4.1±0.9XY |
|  | Straw | 9.4±1.1a | 9.4±1.3A | 5.4±0.9x | 6.4±0.4X |
|  | P | 3.9±0.2b | 5.5±0.9B | 3.6±0.4y | 2.2±0.4Y |
|  | P + straw | 8.5±1.1a | 7.9±0.8AB | 7.3±1x | 9±2X |
| *V. unguiculata* | Control | 3.9±0.5b | 5.8±0.9A | 4.3±0.8xy | 5±0.8X |
|  | Straw | 9.2±1a | 7±0.4A | 5.9±1x | 7.5±0.6X |
|  | P | 3.8±0.5b | 5±0.7A | 3.5±0.7y | 1.7±0.2Y(*) |
|  | P + straw | 7.7±0.7a | 6.3±1A | 6.7±0.8x | 6.2±0.7X |

Note: Control: no fertilizer P or straw; Straw: no P but with straw addition; P: fertilizer P at 100 mg P kg^-1^ soil; P + straw: fertilizer P at 100 mg P kg^-1^ soil together with straw addition. D24: 24 days after straw addition, and D40: 40 days after straw addition. D24: 24 days after straw addition, and D40: 40 days after straw addition. Data are means ± SE (n = 4). For each crop species and a given parameter, the lowercase letters denote significant differences (*p* ≤0.05) among treatments without P or straw, with straw but no fertilizer P, with fertilizer P but no straw, and with fertilizer P and straw at day 24, and the capital letters denote significant differences (*p* ≤0.05) among the four treatments at day 40. For each parameter, t-tests were run to detect significant difference between days 24 and 40: *, p ≤0.05; **, p ≤0.01. Absence of asterisks denotes no difference between days 24 and 40.

**Table S7** Effects of straw addition and P fertilization on abundance of *phoD* and *pqqC* genes in the bulk soil in which *B. chinensis*, *S. lycopersicum*, *L. sativa*, and *V. unguiculata* at days 24 and 40.

| Treatments | | *phoD* gene  (× 10^6^ copies g^-1^ soil) | | *pqqC* gene  (× 10^6^ copies g^-1^ soil) | |
| --- | --- | --- | --- | --- | --- |
|  |  | D24 | D40 | D24 | D40 |
| *B. chinensis* | Control | 5.2±0.8b | 6.4±0.6A | 1.8±0.4y | 3.1±0.6Y |
|  | Straw | 11±1a | 7.8±1A | 2.8±0.3y | 3.4±0.4Y |
|  | P | 5.9±0.7b | 5.4±0.9A | 1.6±0.3y | 4.8±0.5XY(**) |
|  | P + straw | 9.3±0.6a | 4.5±1.3A(*) | 5.1±0.9x | 5.9±0.6X |
| *S. lycopersicum* | Control | 3.8±0.8b | 9.2±1.5A(*) | 2.5±0.4y | 5.1±1.2Y |
|  | Straw | 11.5±2.1a | 10±1.2A | 3.3±0.3y | 7.6±1.2XY(*) |
|  | P | 6±0.7b | 6.8±0.7A | 2±0.4y | 7.6±0.9XY(**) |
|  | P + straw | 11.8±1.9a | 9.9±1.4A | 6.7±0.8x | 9.5±1.1X |
| *L. sativa* | Control | 5±0.5c | 4.9±0.9A | 2.1±0.3y | 3.1±0.7Y |
|  | Straw | 9.6±0.8a | 7.2±0.9A | 2.9±0.3y | 4±0.5Y |
|  | P | 7.1±0.3b | 6.5±0.8A | 1.3±0.1z | 4.4±0.7Y(**) |
|  | P + straw | 8.4±1.7ab | 6.9±1.6A | 4.8±0.9x | 7.1±1.1X |
| *V. unguiculata* | Control | 6.1±0.9b | 7±1.2A | 3.1±0.5y | 3.2±0.9Y |
|  | Straw | 10±1a | 8±1A | 3±0.4y | 10.9±0.6X(*) |
|  | P | 5.9±1.1b | 8.4±1.3A | 2.2±0.3y | 4.1±0.5Y(*) |
|  | P + straw | 8.4±1.1ab | 10.5±1.5A | 7.5±0.7x | 10±1.7X |

Note: Control: no fertilizer P or straw; Straw: no P but with straw addition; P: fertilizer P at 100 mg P kg^-1^ soil; P + straw: fertilizer P at 100 mg P kg^-1^ soil together with straw addition. D24: 24 days after straw addition, and D40: 40 days after straw addition. D24: 24 days after straw addition, and D40: 40 days after straw addition. Data are means + SE (n = 4). For each crop species and a given parameter, the lowercase letters denote significant differences (*p* ≤0.05) among treatments without P or straw, with straw but no fertilizer P, with fertilizer P but no straw, and with fertilizer P and straw at day 24, and the capital letters denote significant differences (*p* ≤0.05) among the four treatments at day 40. For each parameter, t-tests were run to detect significant difference between days 24 and 40: *, p ≤0.05; **, p ≤0.01. Absence of asterisks denotes no difference between days 24 and 40.

**Table S8** Effects of straw addition and P fertilization on C and P in microbial biomass in the bulk soil in which *B. chinensis*, *S. lycopersicum*, *L. sativa*, and *V. unguiculata* at days 24 and 40.

| Treatments | | MBC (mg kg^-1^ soil) | | MBP (mg kg^-1^ soil) | |
| --- | --- | --- | --- | --- | --- |
|  |  | D24 | D40 | D24 | D40 |
| *B. chinensis* | Control | 89±16c | 707±29A(***) | 16±3z | 19±2Y |
|  | Straw | 662±27a | 551±22B(*) | 39±2y | 23±3Y(**) |
|  | P | 369±52b | 328±21C | 27±4y | 42±4X(*) |
|  | P + straw | 631±52a | 726±20A | 62±6x | 41±4X(*) |
| *S. lycopersicum* | Control | 116±7d | 396±30B(***) | 30±2z | 24±3Y |
|  | Straw | 470±56b | 607±46A | 43±2y | 28±2Y(**) |
|  | P | 331±26c | 382±18B | 47±8y | 22±2Y(*) |
|  | P + straw | 650±38a | 672±33A | 76±5x | 54±4X(*) |
| *L. sativa* | Control | 125±12d | 267±6C(***) | 29±2z | 31±4Z |
|  | Straw | 452±36b | 430±57AB | 40±3y | 30±4Z |
|  | P | 274±24c | 338±25BC | 28±2z | 45±3Y(**) |
|  | P + straw | 641±62a | 563±36A | 57±2x | 73±5X(**) |
| *V. unguiculata* | Control | 128±11c | 427±58(**) | 25±2z | 36±4Y(**) |
|  | Straw | 330±24b | 542±9(***) | 42±6y | 38±3Y |
|  | P | 291±22b | 531±50(**) | 29±4yz | 42±3Y(*) |
|  | P + straw | 883±59a | 586±34(**) | 60±4x | 84±8X(**) |

Note: Control: no fertilizer P or straw; Straw: no P but with straw addition; P: fertilizer P at 100 mg P kg^-1^ soil; P + straw: fertilizer P at 100 mg P kg^-1^ soil together with straw addition. D24: 24 days after straw addition, and D40: 40 days after straw addition. D24: 24 days after straw addition, and D40: 40 days after straw addition. MBC: microbial biomass C; and MBP: microbial biomass P. Data are means ± SE (n = 4). For each crop species and a given parameter, the lowercase letters denote significant differences (*p* ≤0.05) among treatments without P or straw, with straw but no fertilizer P, with fertilizer P but no straw, and with fertilizer P and straw at day 24, and the capital letters denote significant differences (*p* ≤0.05) among the four treatments at day 40. For each parameter, t-tests were run to detect significant difference between days 24 and 40: *, p ≤0.05; **, p ≤0.01; ***, p ≤0.001. Absence of asterisks denotes no difference between days 24 and 40.
